# “Design characteristics of the CORRONA CERTAIN study: a comparative effectiveness study of biologic agents for rheumatoid arthritis patients”

**DOI:** 10.1186/1471-2474-15-113

**Published:** 2014-04-01

**Authors:** Dimitrios A Pappas, Joel M Kremer, George Reed, Jeffrey D Greenberg, Jeffrey R Curtis

**Affiliations:** 1 Columbia University College of Physicians and Surgeons, New York, NY, USA; 2 Consortium of Rheumatology Researchers of North America (CORRONA), Southborough, MA, USA; 3 Department of Medicine, Albany Medical Center & The Center for Rheumatology, Albany, NY, USA; 4 University of Massachusetts, Worcester, MA, USA; 5 New York School of Medicine, New York, NY, USA; 6 Division of Clinical Immunology and Rheumatology, University of Alabama at Birmingham, Birmingham, AL 35294-7201, USA

**Keywords:** Rheumatoid arthritis, Registry, Cohort, Epidemiology, Comparative effectiveness, Safety, Biologics

## Abstract

**Background:**

Comparative effectiveness research has recently attracted considerable attention. The *C*omparative *E*ffectiveness *R*egistry to study *T*herapies for *A*rthritis and *I*nflammatory Co*n*ditions (CERTAIN) is an ongoing prospective cohort study of adult patients with Rheumatoid Arthritis (RA).

**Methods/Design:**

CERTAIN uses the existing Consortium of Rheumatology Researchers of North America (CORRONA) network of participating private and academic sites in order to recruit patients fulfilling the 1987 ACR criteria that have at least moderate disease activity. Patients starting or switching biologic agents either anti-TNF therapy or a non anti-TNF biologic are eligible for enrollment, depending on the treatment selected by their physician. Enrollment is expected to be completed by March of 2014, and 2711 patients will participate in the study. As of October 7th 2013, 2234 patients have been enrolled. Patient visits and laboratory blood work are mandated every three months for one year. Safety data is collected through one year and beyond. The primary comparative effectiveness endpoint is attainment of low RA disease activity at one year among patients who have been exposed to at least one prior TNF-α inhibitor agent prior to enrollment. Multiple secondary effectiveness and safety endpoints will be addressed by investigating the entire population enrolled (naïve and biologic experienced).

**Discussion:**

The unique design features of CERTAIN will inform comparative effectiveness and safety questions for choosing biologic agents for the management of RA.

## Background

Considerable attention and funding has recently been allocated to comparative effectiveness research (CER). In the U.S., for example, the American Recovery and Reinvestment Act (ARRA) devoted approximately 1 billion dollars in support of such studies in 2009 [1].

According to the Institute of Medicine, “The purpose of CER is to assist consumers, clinicians, purchasers, and policy makers to make informed decisions that will improve health care at both the individual and population levels” [2]. CER cannot only compare existing therapies in widespread use but also has the potential to establish standards and a mechanism by which newly available medications can be evaluated and compared to standard therapies. It may facilitate the creation of a more demanding scientific and medical community culture by which promotion of innovation in drug discovery will be encouraged, as opposed to the production of cloned “me too” therapeutics lacking robust evidence of superiority against existing medications [2].

### Biologic agents in rheumatoid arthritis and the need for CER in rheumatology

Biologic agents have revolutionized the treatment of rheumatoid arthritis (RA) over the last decade. Their efficacy and safety has been clearly demonstrated in the setting of a multitude of randomized controlled trials (RCTs). Results for each agent are broadly comparable across all outcome domains including ACR (American College of Rheumatology) and EULAR (EUropean League Against Rheumatism) responses, improvement in quality of life, and arrest or reversal of radiologic damage [3-5]. With the approval of 2 additional TNF inhibitors (golimumab and certolizumab) and an IL-6 receptor inhibitor (tocilizumab) the current therapeutic armamentarium contains 9 biologic agents for the treatment of patients with inflammatory arthritis. However, these medications were studied and approved against comparator arms containing placebo, which may not have significant relevance to clinical practice. Despite regulatory requirements for drug approval, showing that a biologic agent is better than placebo does not provide a relevant context with which to choose among the available treatment options for RA patients.

Moreover, the magnitude of benefit of biologic agents in typical RA patients seen in every day practice – as opposed to clinical trial participants – has been less clearly demonstrated, especially for patients with mild or moderate RA disease activity or those with high burdens of medical comorbidities. These individuals would generally not qualify to participate in a clinical trial; indeed, only a minority of patients seen in clinical practice would qualify for a clinical trial [6-9].

Similar limitations in generalizability, and in understanding long term safety, are not available for industry-conducted head-to-head randomized control trials (RCT) comparing biologics, especially among patients with prior biologic exposure.

Lastly, the estimated per person cost of a typical biologic agent ranges between $15,000-22,000 per year, or more [10,11]. While this may be justified by the extent of clinical benefit they offer to patients, the evidence from CER studies could be used to better inform cost-effectiveness considerations regarding the use of biologics in RA. The perception that all biologics “are overall the same” may be contributing to keeping cost high and at roughly comparable levels. As an example of prior comparative effectiveness research that has impacted clinical practice, it is now known that the first line anti-hypertensive treatment can be an inexpensive but effective thiazide diuretic instead of a more costly angiotensin converting enzyme inhibitor. If not for the ALLHAT study –the landmark CER study which demonstrated this outcome- it is rather doubtful that the prescribing habits of internists treating hypertension would have been altered to promote greater use of the less costly treatment option [2,12].

### The CORRONA network and registry. Mission, history, governance and funding

The Consortium of Rheumatology Researchers of North America (CORRONA) was founded in 2001. The CORRONA registry collects longitudinal, “real-world” data from patients and their treating physicians. At the time of this writing, data on 45,229 patients with rheumatologist-diagnosed inflammatory arthritis, including 38,776 patients with RA, have been collected. The CORRONA participating site network is comprised of more than 100 private and academic practices across 42 states within the United States, with more than 350 rheumatologists contributing data. All geographic regions in the continental United States are represented and there are no age, racial, disease activity or other restrictions to patient participation in the registry. As of October 7th 2013, CORRONA’s database included information about 290,020 patient visits, 119,955 patient years of follow-up observation time, with a mean time of patient follow up of 3.4 years (median 2.6 years).

At each CORRONA registry visit patients and physicians record data on disease severity and activity, RA and other medications, adverse events, quality of life, selected laboratory and imaging results, and socio-demographic information.

By way of providing a brief review of CORRONA’s history, an independent database collecting data from both rheumatologists and patients with inflammatory arthritis did not exist in the US at the time the organization was founded, and CORRONA aspired to fill this gap. A group of experienced academic and private rheumatologists founded and now serve on CORRONA’s board of directors which is entirely responsible for its governance and its scientific oversight. Operational needs are covered with funding predominantly derived from the pharmaceutical industry which may submit queries for data analysis but does not have access to CORRONA’s raw data. Instead all queries are evaluated and analyses are performed by academic-based biostatisticians and epidemiologists. Query results are generated and provided as summary reports to the requesting pharma company [13]. Pharmaceutical companies may submit an abstract or manuscript from the obtained data, but must follow CORRONA’s publication and authorship policies. A CORRONA investigator serves as the lead author, and has final authority on all elements of the published work.

CORRONA’s successes are exemplified by its contribution of multiple publications [7-21] in high-yield scientific journals. To date, CORRONA has collected data at office visits, as often as every 3 months unless a biologic agent is started, in which case more frequent data collection is allowable. For routine care in the absence of a change of these drugs, visits have occurred at a mean interval of 4.5 months. Unlike in CERTAIN, the CORRONA core registry does not mandate specific laboratory values, and thus the labs collected by the CORRONA registry reflect what is felt to be appropriate by the treating rheumatologist in the course of routine clinical care. Thus, certain values such as acute phase reactants are sometimes absent.

### The *C*omparative *E*ffectiveness *R*egistry to study *T*herapies for *A*rthritis and *I*nflammatory Co*n*ditions (CERTAIN)

In an attempt to expand the scope of clinical data, and to focus the scientific yield on comparative effectiveness, CORRONA launched the CERTAIN study in late 2010. CERTAIN is a prospective, non-randomized cohort study of adult patients with RA fulfilling the 1987 ACR criteria, having at least moderate disease activity defined by a clinical disease activity index (CDAI) score >10 who are starting or switching biologic agents [22]. As of October 7th 2013, 2234 patients were enrolled across 43 participating academic and private rheumatology practices.

## Methods/Design

The CERTAIN Sub-study has been designed to systematically collect and compare the effectiveness and safety of biologic medications (i.e. anti-TNF therapy, abatacept, rituximab, tocilizumab). The decision to recruit a patient into CERTAIN is made during a routine patient visit when a treating rheumatologist determines that a biologic agent for RA should be started. Even though the primary endpoint is to investigate comparative effectiveness among patients who have been exposed to at least one TNF-α inhibitor, CERTAIN will also enroll naïve to biologic agents patients in order to address multiple additional secondary endpoints and inform comparative safety research. For these secondary analyses of biologic-naïve patients, and in contrast to the main hypothesis to be examined by CERTAIN, it is likely that the anti-TNF and non anti-TNF groups would not be directly compared to one another given the anticipated small numbers and substantial heterogeneity in biologic-naïve patients initiating non anti-TNF therapy. Patients who initiate non anti-TNF agents as a first line biologic might be expected to have comorbidities (e.g. heart failure, cancer) that would make them dissimilar to new anti-TNF users.

Patients must fulfill the 1987 ACR criteria for RA and have moderate disease activity (i.e. CDAI > 10) in order to be eligible for participation. All existing or new CORRONA patients will be given the opportunity to participate. The first visit functions as the screening visit, during which patient’s consent is obtained and the process of insurance approval for the biologic to be started is initiated. After insurance approval is obtained, the patient returns for a baseline visit, and then for mandated follow up visits every three months through 1 year (i.e. baseline and 3,6,9,12 months follow up visits). Thus, the visit schedule of CERTAIN mimics that of an open-label controlled trial (RCT) with required follow visits at 3 month intervals. All biologic agents prescribed are approved by the Food and Drug Administration and the choice of which biologic to be initiated is entirely at the discretion of the prescribing physician.

The full set of data collected by the CORRONA registry are collected at each CERTAIN visit and in addition, mandated laboratory tests are performed, as indicated in Table 1. In addition, patients are requested to provide a sample of blood for DNA extraction and genotyping for future pharmacogenetics research. Whole blood for gene expression studies, as well as serum and plasma, is stored for future biomarker studies. All blood samples are shipped directly from the participating sites on the day of blood draw to a central laboratory where analyses are performed. The patients are reimbursed for their inconvenience, and physicians are provided with the results of some of the clinical lab tests at no cost to the patients or their insurance in order to facilitate clinical care and avoid redundant testing and phlebotomy.

**Table 1:** Clinical and laboratory assessments during CERTAIN

	**Baseline**	**Follow up visits**
	**0 Months**	**3, 6, 9, 12 Months**
**Assessments**		
Demographics (date of birth, sex, address, email)	X	
Medication exposures (including biologics)	X	X
Tender & Swollen Joint Count (0–28)	X	X
Physician Global Disease Activity (100 mm VAS)	X	X
Patient Global Disease Activity (100 mm VAS)	X	X
Patient Pain (100 mm VAS)	X	X
Patient Fatigue (100 mm VAS)	X	X
EuroQol 5D	X	X
HAQ DI	X	X
**Laboratory assessments (run at a central laboratory)**		
Complete blood count (with automated differential)	X	X
Rheumatoid factor and isotypes	X	
Anti- cyclic citrullinated peptide antibody	X	
High-sensitivity C–reactive protein (hsCRP)	X	X
Immunoglobulin panel (IgM, IgG, IgA)	X	X
Traditional non fasting lipoprotein analyses (HDL, Total cholesterol, Triglycerides)	X	X
Direct quantitative LDL (fasting specimens not required)	X	X
Serum, plasma, whole blood (RNA)	X	X*
AST ALT, Albumin, Total and direct Bilirubin, BUN, Creatinine, Glucose, Calcium, Serum uric acid, CK, LDH	X	X
DNA (optional component)	X	

*Abbreviations*: *VAS* visual analog scale, *CRP* C-reactive protein, *HAQ* health assessment questionnaire, *DI* disability index, *HDL* high density lipoprotein, *LDL* low density lipoprotein, *RNA* ribonucleic acid, *AST* aspartate aminotransferase, *ALT* alanine aminotransferase, *BUN* blood urean nitrogen, *CK* creatinine kinase, *LDH* lactate dehydrogenase, *DNA* deoxyribonucleic acid.

*Collected at baseline, 3 and 6 month follow up visits.

### Quality assurance and quality control procedures for data collection

Investigators and staff at the 43 academic and private practices participating in CERTAIN completed a comprehensive online and on-site training on the study protocol prior to study initiation. The training materials were prepared and delivered by CORRONA personnel and were tailored to individuals’ roles (e.g. investigators, research coordinator). Ongoing quality control processes are in place to ensure high quality and rigorous data collection, overseen by a dedicated team. Study data are monitored via regular in-person site visits in order to ensure completeness and accuracy and to help sites resolve open queries.

### Recruitment targets and ratios

CERTAIN is intended to focus on comparative effectiveness of established and newly approved biologic treatments for RA for patients who have failed to therapy with at least one TNF-α inhibitor prior to enrollment.

Given the well-characterized profile of existing biologics for RA, uptake of newer RA treatments is sometimes slow. For that reason, and to maximize statistical power for comparative analyses, CERTAIN established a goal to recruit anti-TNF and non anti-TNF therapies in an approximately 1:1 ratio. Perturbation of this ratio in up to a 3:2 ratio (in either direction) at each study site is permissible. In the case of exceeding the enrollment ratio, sites with extreme perturbations beyond the 3:2 limit will be instructed to temporarily not enroll patients starting biologics in the study arm in excess.

Individual agents within the anti-TNF and non anti-TNF categories are not differentiated in the primary analysis, nor are treatments within each category mandated. Treatment selection is fully under the control of the physician and patients are not randomized. The decision to not randomize patients was made in light of a dearth of evidence regarding the optimal treatment strategy for patients who fail one or more anti-TNF inhibitors. Given this state of relative equipoise in the decision to switch to another anti-TNF agent or to change to a biologic with a different mechanism of action, confounding in the form of channeling patients to specific medications is likely to be less problematic than comparisons of biologics vs. non-biologic DMARDs. Patients who do not qualify for CERTAIN based upon disease activity criteria or the enrollment ratio are recruited to the CORRONA “core” registry.

**Figure 1:**
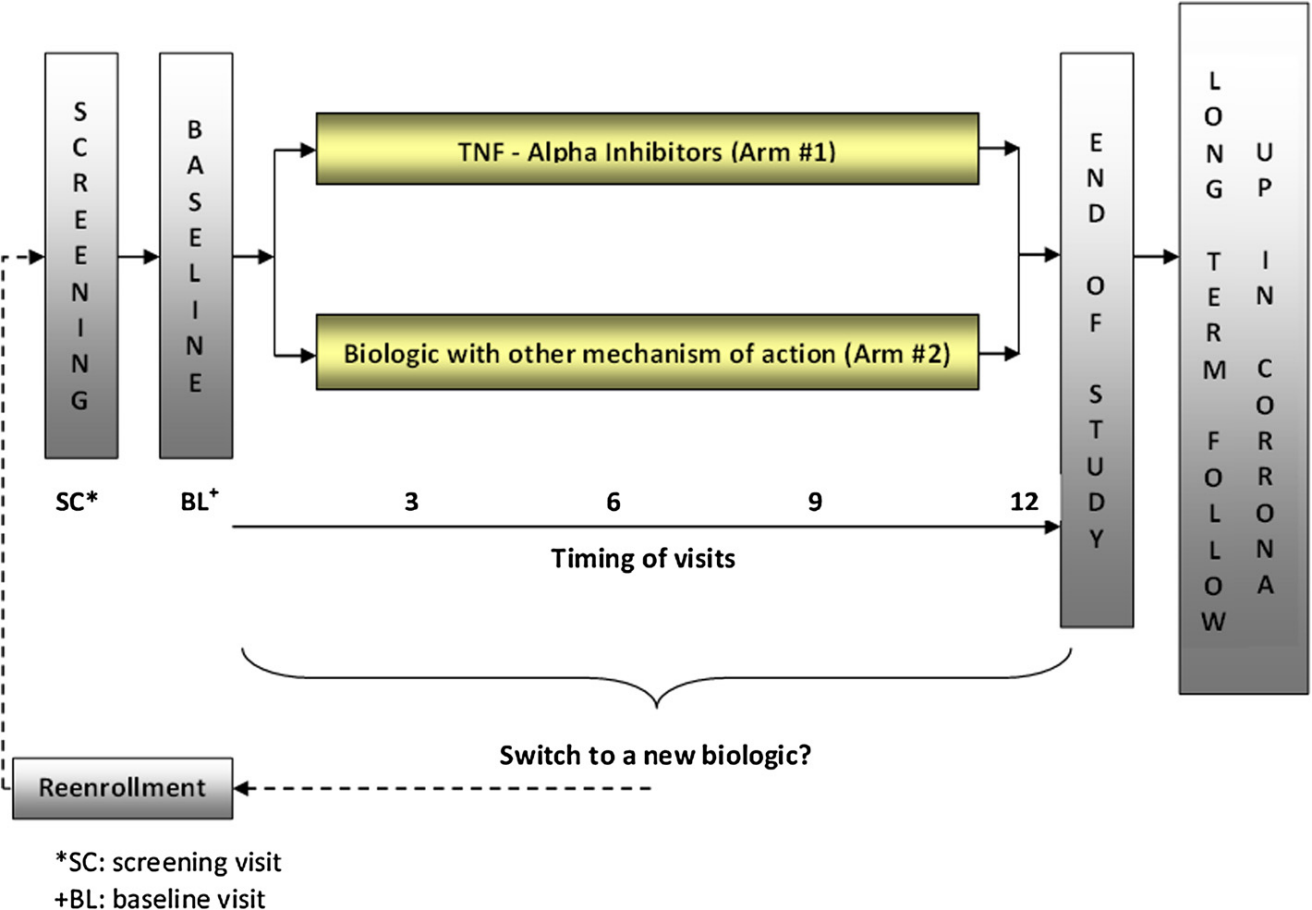
Schematic of CERTAIN study design.

In order to reflect a ‘real world’ effectiveness setting, patients are permitted to change or discontinue biologic therapies at their physician’s discretion. If they start a new biologic, however, this action requires a new study visit at that time. This new study visit (i.e. an ‘early termination’ visit) ends follow-up time for the first drug and can define a new screening visit for the next biologic if the physician and patient so chooses. Patients are allowed to contribute multiple sets of observations if they initiate different biologics over the study period; if they do so, a new ‘baseline’ visit is established. Participants will continue to be followed longitudinally in the CORRRONA ‘core’ registry protocol after the completion of the one-year CERTAIN study. This important feature allows for long term follow-up for safety and effectiveness. The above are summarized in Figure 1.

### Primary endpoint and covariates of interest

The primary endpoint of CERTAIN is attainment of low disease activity (LDA) one year after starting or switching biologic agents and will be assessed only among patients who have previously been treated with one or more anti-TNF-α therapies. Patients who are biologic naïve at enrollment will contribute data to secondary analyses. LDA is defined as a CDAI ≤ 10. Although enrollment criteria and LDA could be defined using DAS28 (disease activity score using 28 joint counts), the DAS28 requires knowing the value of the acute phase reactant result in real time, which is generally not feasible. For that reason, the CDAI was chosen as the RA disease criterion for enrollment and primary outcome measure, given the high correlation between CDAI and DAS28 [22]. Using a dichotomous outcome (response vs non response) for the primary outcome has limitations, and any single threshold for considering a patient to be a ‘responder’ is arbitrary. However, LDA was chosen to reflect a clinically meaningful endpoint that would likely result in a patient continuing on that therapy. Thus, LDA is considered as a proxy outcome for a ‘responder’. Improvement in disease activity (as a continuous measure), controlling for baseline disease activity, will be examined in secondary effectiveness analyses. Consistent with our goals of evaluating effectiveness rather than efficacy, persons who switch to a new biologic (i.e. non-persistence), either for reasons of efficacy or safety, will be considered a non-responder. As additional secondary endpoints, DAS28 and other clinical outcomes (e.g. Health Assessment Questionnaire, or HAQ), ACR response, EULAR response, DAS remission, CDAI remission) at various time points also will be examined.

Allocation to specific biologic treatment is not randomized in CERTAIN. In order to overcome this potential source of confounding whereby patients with certain characteristics might be channeled to particular therapies, analytic adjustment will be performed to maximize the validity of treatment comparisons by reducing confounding, and improve precision. Propensity scores for receipt of anti-TNF vs. non anti-TNF therapy will be constructed based upon a priori and empirically-derived covariates that will include number of prior biologic used, concomitant MTX, concomitant glucocorticoid use/dose, duration of RA, and reason for previous biologic discontinuation (primary vs. secondary non-response vs. safety/tolerability vs. other). Treatment episodes for patients in the non-overlapping distributions of the propensity score will be trimmed (expected to be < 5% of observations removed for this reason, based upon preliminary examination), and multivariable adjustment will be used for the resulting main analysis population to control for relevant confounding. Numerous other potential confounders and effect modifiers including socio-demographic, anthropometric and disease specific characteristics will be controlled for as needed. As one example of potentially important covariates, it is possible that patients’ health insurance may affect the selection of biologic agents initiated in CERTAIN, and it may not be possible to fully characterize these payor influences on medication selection. Nevertheless, while this might raise concern for potential confounding, selection of specific biologics over another that are dictated solely by insurance type rather than patient characteristics may reduce confounding and bias due to channeling. Additionally, clustering by physician practice also will be accounted for in the analytic approach. Appropriate statistical techniques (e.g. mixed models, or generalized estimating equations) will be applied to account for patients who contribute multiple treatment episodes to the analysis. Based upon power calculations for the main hypothesis to demonstrate at least a 10% difference in the proportion of patients achieving LDA at 1 year between anti-TNF and non anti-TNF treated patients, CERTAIN plans to recruit approximately 2711 eligible patients over a three-year period.

### Comparative safety in CERTAIN

The Institute of Medicine (IOM) has proposed a definition for comparative effectiveness that encompasses the domain of safety [23]. For that reason, beyond determining the comparative clinical effectiveness of various biologics for controlling disease activity, it is critical to better understand the risks of the biologics for serious adverse events (SAEs), if any such risks differ among agents, and if there are patient populations for which the risks associated with these agents are particularly high. Serious adverse events (SAEs) are of high interest and are a key part of comparative safety that will be evaluated within CERTAIN. Pre-specified SAEs of high interest include serious infections, myocardial infarction, stroke, malignancy, GI perforation, anaphylaxis, liver failure, bleeding, and demyelinating events. The FDA definition of serious adverse event also will apply (http://www.fda.gov/safety/medwatch/howtoreport/ucm053087.htm). Key criteria include death, hospitalization, life-threatening event and disability or permanent damage.

Initial case ascertainment for each of these events predominantly relies on reports from physicians and patients and is obtained from the CERTAIN case report forms. Following reporting by a physician, the CERTAIN data coordinating center requests the site to complete a short form to confirm the event and to obtain additional clinical details. These confirmation forms are outcome specific. Concurrent with the request to complete the confirmation form, medical records from the hospitalization or other pertinent sources of data (e.g. pathology reports for malignancies) are requested. Both the confirmation form and medical records are de-identified and faxed to the CERTAIN data coordinating center.

For each SAE, the confirmation form and associated medical records are sent to a group of physicians who centrally adjudicate all events according to pre-specified criteria. These physicians are blinded to drug exposure status to avoid bias. The classification criteria for SAE adjudication use standardized criteria whenever possible. For example for serious infections, the criteria system used has been described [21]. All reported events are classified according to their level of certainty (e.g. confirmed, probable, possible, unlikely).

Although medical record retrieval from the individual CERTAIN sites is high (historically, approximately 90%), CERTAIN has the ability to request medical records directly from healthcare facilities. This is facilitated by the CERTAIN investigators at University of Alabama (UAB) functioning as an ‘honest broker’ to maintain CERTAIN personal identifiers. Researchers at UAB therefore have the ability to request medical records directly from hospitals or physician offices. If necessary, UAB researchers also have the ability to contact CERTAIN participants directly to obtain updated or facility-specific medical record release forms, thus ensuring high rates of medical record retrieval. Through this mechanism, patients can also be contacted (if necessary) to obtain updated medical record release forms, or for other appropriate purposes (e.g. conducting optional patient-targeted surveys by email, Internet, phone, or mail). Patients consent to both data collection and these additional features. The study is governed by both a central institutional review board (IRB) [the New England IRB] as well as local and university-based IRBs if required at individual sites.

An independent mechanism to ensure completeness of SAE case ascertainment and mortality is also available. CERTAIN participants are consented and asked to provide identifying data that can be used to link to administrative claims databases (e.g. Medicare, commercial insurance) and other national data sources (e.g. the National Death Index). Approximately 40% of RA patients in CERTAIN are anticipated to be linkable to Medicare/Medicaid administrative claims data. Although there is some lag in the availability of the administrative data, the linkages between the CERTAIN clinical data and administrative claims databases allow confirmation of the completeness of the physician-reported SAEs. In this way, CERTAIN has a method to externally validate the absolute incidence rates for the various outcomes of interest and also evaluate the generalizability of CERTAIN participants and their characteristics (e.g. co-morbidities) compared to non-enrolled individuals (e.g. other RA patients treated in geographically similar physician practices with the same health insurance) [24]. The administrative data linkage will also allow for examination of a number of other important outcomes (e.g. medication adherence, costs and health economics).

### Genomics, genetics and comparative effectiveness

Concerns have been expressed that comparative effectiveness research may not be applicable to individual patients with unique genetic backgrounds [25], and a need for bridging the “chasm” between CER and personalized medicine has been recognized [26]. It has been posited that both CER and genomic medicine will complement each other as long as genome-based perspectives are incorporated in the design of CER studies [26].

In this context, DNA is collected at the time of the baseline CERTAIN visit and will be used for candidate gene and genome-wide analyses to predict response to treatment or susceptibility to adverse events while on treatment with biologics. The DNA collected during this study is creating a rich genomic repository which will allow a multitude of hypotheses to be tested with adequate power and sample sizes.

### Enrollment status and baseline data

CERTAIN is currently enrolling patients throughout the U.S. As of October 7th 2013, 2234 patients had been enrolled. Detailed enrollment data for these participants are available. Basic demographic, disease activity characteristics and distribution of comorbities are presented in Tables 2 and 3. Table 2 shows data for the treatment episodes of TNF-α inhibitor experienced patients that will be included in the primary analysis. As shown, RA disease characteristics were generally well balanced between the two treatment groups. Based upon comparison of the standardized absolute mean difference between anti-TNF and non anti-TNF users, the characteristics that were most different included RA disease duration (median 6 vs. 8 years), median DAS28CRP (4.7 vs. 5.0), and daily prednisone dose (5 vs. 7 mg). Most other differences were small. For example, mean disease activity by CDAI was comparable: 27 (anti-TNF treated) vs. 28 (non anti-TNF treated). Likewise, the prevalence of key comorbidities was similar between anti-TNF and non anti-TNF patients.

**Table 2:** CERTAIN patients who have been exposed to at least 1 TNF-α inhibitor (population used for primary comparative effectiveness analyses)

	**All**	**Anti-TNF agents initiators**	**Non-anti-TNF agents initiators**	**Standardized absolute mean difference**
**Total, n**	**1321**	**558**	**763**	
Age (median, IQR), years	57 [47,65]	56 [47,64]	57 [48,66]	0.137
Female, n (%)	1037 (79.9)	434 (79.1)	603 (80.5)	0.036
Caucasian, n (%)	1091 (83.9)	446 (81.1)	645 (85.9)	0.129
RA disease duration (median, IQR), years	7 [3,14]	6 [3,12]	8 [4,15]	0.213
CDAI (median, IQR)	28 [21,38]	27 [20,38]	28 [21,38]	0.072
DAS28-CRP (median, IQR)	4.9 [4.2,5.6]	4.7 [4,5.4]	5.0 [4.3,5.7]	0.259
Biologic Monotherapy, n (%)	436 (33)	188 (33.7)	248 (32.5)	0.025
Concurrent DMARDs, n (%)	885 (67)	370 (66.3)	515 (67.5)	0.025
MTX only, n (%)	586 (44.4)	258 (46.2)	328 (43)	0.065
MTX dose (med, IQR) mg	20 [15,20]	20 [15,20]	20 [15,20]	0.018
MTX plus other DMARDs, n (%)	95 (7.2)	40 (7.2)	55 (7.2)	0.002
Leflunomide only, n (%)	78 (5.9)	28 (5)	50 (6.6)	0.066
Sulfasalazine only, n (%)	18 (1.4)	4 (0.7)	14 (1.8)	0.100
Hydroxychloroquine only, n (%)	53 (4)	20 (3.6)	33 (4.3)	0.038
Concomitant prednisone, n (%)	460 (34.8)	198 (35.5)	262 (34.3)	0.024
Prednisone dose (median, IQR) mg/day	5 [5,10]	5 [5,10]	7 [5,10]	0.155
No use, n (%)	877 (66.4)	363 (65.1)	514 (67.4)	0.049
Prednisone <5 mg, n (%)	53 (4)	18 (3.2)	35 (4.6)	0.070
Prednisone 5- <10 mg, n (%)	202 (15.3)	100 (17.9)	102 (13.4)	0.125
Prednisone ≥ 10 mg, n (%)	189 (14.3)	77 (13.8)	112 (14.7)	0.025
Number of prior biologics exposed to (median, IQR)	1 [1,2]	1 [1,2]	1 [1,3]	0.427
Number of prior non-biologic DMARDs exposed to (median, IQR)	2 [1,3]	2 [1,2]	2 [1,3]	0.272
Biologic started at enrollment, n (%)				
Adalimumab	122 (9.2)	122 (21.9)	N/A	N/A
Infliximab	127 (9.6)	127 (22.8)	N/A	N/A
Etanercept	101 (7.6)	101 (18.1)	N/A	N/A
Golimumab	71 (5.4)	71 (12.7)	N/A	N/A
Certolizumab	137 (10.4)	137 (24.6)	N/A	N/A
Rituximab	125 (9.5)	N/A	125 (16.4)	N/A
Abatacept	334 (25.3)	N/A	334 (43.8)	N/A
Tocilizumab	304 (23)	N/A	304 (39.8)	N/A
Co-morbidities, n (%)				
History of Cardiovascular disease*	104 (7.9)	42 (7.5)	62 (8.1)	0.022
History of Hypertension	333 (31.9)	144 (33.6)	189 (30.7)	0.063
History of Diabetes Mellitus	102 (10)	45 (10.8)	57 (9.5)	0.042
Hyperlipidemia (Defined as: Total cholesterol > 240 mg/dL at baseline visit)	156 (13)	70 (13.6)	86 (12.5)	0.032
History of Malignancy (includes non -melanoma skin cancers)	100 (7.6)	41 (7.3)	59 (7.7)	0.015

Note: Standardized Absolute Mean Differences of 0.1 or less were considered clinically unimportant.

*Abbreviations*: *IQR* interquartile range, *MTX* methotrexate, *CDAI* clinical disease activity index, *DAS28_CRP* disease activity score with 28 joint counts and CRP (C– reactive protein) as the inflammatory marker.

*History of cardiovascular disease included: cardiac revascularization procedures, ventricular arrhythmias, cardiac arrests, myocardial infarctions, acute coronary syndromes, unstable angina, coronary artery disease, congestive heart failure, stroke, transient ischemic attacks.

(Data for patients enrolled as of October 7th 2013).

**Table 3:** CERTAIN patients not included in the primary analysis population because they had not previously received anti-TNF therapy at the time of initiation of a new biologic

	**All**	**Anti-TNF agents initiators**	**Non-anti-TNF agents initiators**	**Standardized absolute mean difference**
**Total, n**	**913**	**762**	**151**	
Age (median, IQR), years	56 [48,65]	55 [47,64]	60 [51,69]	0.349
Female, n (%)	675 (77.2)	563 (76.9)	112 (78.9)	0.047
Caucasian, n (%)	708 (80.8)	596 (81.2)	112 (78.9)	0.058
RA disease duration (median, IQR), years	2 [1,7]	2 [1,6]	4 [1,11]	0.294
CDAI (median, IQR)	27 [20,37]	26 [20,36]	28 [20,38]	0.115
DAS28-CRP (median, IQR)	4.8 [4.1,5.5]	4.9 [4.1,5.5]	4.6 [3.9,5.4]	0.114
Biologic Monotherapy, n (%)	221 (24.2)	178 (23.4)	43 (28.5)	0.117
Concurrent DMARDs, n (%)	692 (75.8)	584 (76.6)	108 (71.5)	0.117
MTX only, n (%)	486 (53.2)	425 (55.8)	61 (40.4)	0.311
MTX dose (med, IQR) mg/week	20 [15,20]	20 [15,20]	20 [15,20]	0.149
MTX plus other DMARDs, n (%)	95 (10.4)	81 (10.6)	14 (9.3)	0.045
Leflunomide only, n (%)	39 (4.3)	29 (3.8)	10 (6.6)	0.127
Sulfasalazine only, n (%)	13 (1.4)	10 (1.3)	3 (2)	0.053
Hydroxychloroquine only, n (%)	33 (3.6)	27 (3.5)	6 (4)	0.023
Concomitant prednisone, n (%)	287 (31.4)	243 (31.9)	44 (29.1)	0.060
Prednisone dose (median, IQR) mg	5 [5,10]	5 [5,10]	5 [5,6]	0.256
No use, n (%)	636 (69.7)	528 (69.3)	108 (71.5)	0.049
Prednisone <5 mg, n (%)	37 (4.1)	27 (3.5)	10 (6.6)	0.140
Prednisone 5- <10 mg, n (%)	134 (14.7)	111 (14.6)	23 (15.2)	0.019
Prednisone ≥ 10 mg, n (%)	106 (11.6)	96 (12.6)	10 (6.6)	0.203
Number of prior biologics exposed to (median, IQR)	0 [0,0]	0 [0,0]	0 [0,0]	0.425
Number of prior non-biologic DMARDs exposed to (median, IQR)	1 [1,2]	1 [1,2]	2 [1,2]	0.343
Biologic started at enrollment, n (%)				
Adalimumab	268 (29.4)	268 (35.2)	N/A	N/A
Infliximab	180 (19.7)	180 (23.6)	N/A	N/A
Etanercept	205 (22.5)	205 (26.9)	N/A	N/A
Golimumab	20 (2.2)	20 (2.6)	N/A	N/A
Certolizumab pegol	89 (9.7)	89 (11.7)	N/A	N/A
Rituximab	15 (1.6)	N/A	15 (9.9)	N/A
Abatacept	100 (11)	N/A	100 (66.2)	N/A
Tocilizumab	36 (3.9)	N/A	36 (23.8)	N/A
Co-morbidities, n (%)				
History of Cardiovascular disease*	71 (7.8)	54 (7.1)	17 (11.3)	0.145
History of Hypertension	203 (28.2)	165 (26.9)	38 (35.8)	0.194
History of Diabetes Mellitus	63 (9)	52 (8.7)	11 (11.2)	0.085
Hyperlipidemia (Defined as: Total cholesterol > 240 mg/dL at baseline visit)	111 (13.5)	92 (13.3)	19 (14.2)	0.025
History of Malignancy (includes non -melanoma skin cancers)	54 (5.9)	45 (5.9)	9 (6)	0.002

*Abbreviations*: *IQR* interquartile range, *CDAI* clinical disease activity index, *DAS28_CRP* disease activity score with 28 joint counts and CRP (C- reactive protein) as the inflammatory marker.

*History of cardiovascular disease included: cardiac revascularization procedures, ventricular arrhythmias, cardiac arrests, myocardial infarctions, acute coronary syndromes, unstable angina, coronary artery disease, congestive heart failure, stroke, transient ischemic attacks.

(Data for patients enrolled as of October 7th 2013).

Table 3 summarizes similar information for the rest of the enrolled population who were biologic naive. Most of these patients (762/913, or 84%) were initiated on anti-TNF therapy. Patients had much earlier RA disease duration (mean = 2 years for the overall group). As might be expected, given that most RA treatment paradigms have recommended initial biologic treatment to start with anti-TNF therapy, the patients receiving non anti-TNF medications as their first biologic were more dissimilar to the anti-TNF users than the population contributing to the main analysis (Table 2).

## Discussion

CERTAIN is a newly-launched RA comparative effectiveness study examining biologic agents currently approved in the U.S. among patients with moderate or high RA disease activity. The established infrastructure of CORRONA is used for patient enrollment, physician participation, collection and storage of data and mandates visits at regular intervals with centralized laboratory evaluations and a robust biospecimen repository for RA patients initiating or switching biologic agents. Safety data will be generated via a robust system of serious adverse event confirmation with adjudication using medical records and linkage with external databases. Enrollment data from the 2234 patients recruited to-date suggest that among those who have been treated with at least one anti-TNF therapy, characteristics of patients initiating their next biologic were relatively well-balanced between treatment groups. The innovative design features of the CERTAIN study will harness the experience of an existing network of dedicated U.S. physicians and sites to better evaluate the comparative effectiveness of biologic DMARDs.

### Key messages

1. CERTAIN will inform effectiveness and safety questions to compare anti-TNF to non anti-TNF biologic agents for the treatment of rheumatoid arthritis.

2. Innovative design elements of CERTAIN will incorporate state of the art methods for comparative effectiveness research.

## Competing interests

Dimitrios A Pappas MD, MPH: Employee of CORRONA, Inc; Honoraria: Novartis. Jeffrey Greenberg, MD, MPH: Grants/Research Support: BMS; Consultant: Novartis; Roche; UCB; Centocor; Genetech; Other: Remuneration as Chief Scientific Officer, CORRONA. George Reed, PhD: Employee of CORRONA, Inc. Joel M Kremer MD: President of CORRONA. Consulting/Honoraria: Amgen, Abbott, BMS, Genentech, Ortho-Biotech, Pfizer, Roche, UCB, Vertex. Grant support: Amgen, Abbott, BMS, Genentech, Ortho-Biotech, Pfizer, Roche, UCB, HGS. Jeffrey R Curtis MD, MS, MPH: Research Grants and/or Consulting: Roche/Genentech, UCB, Janssen, Abbvie, BMS, Amgen, CORRONA, Pfizer.

## Authors’ contributions

All coauthors were responsible for the design and conduct of the study, data collection, analysis, and drafting and final approval of the manuscript. The development of the manuscript and its final submission was not subject to sponsor approval. All authors read and approved the final manuscript.

## Pre-publication history

The pre-publication history for this paper can be accessed here:

http://www.biomedcentral.com/1471-2474/15/113/prepub
